# Emotional Comprehension Is Not Related to Duration of Distress from Daily Life Events

**DOI:** 10.3390/ijerph18020459

**Published:** 2021-01-08

**Authors:** Jaume Vives, Cristina Morales, Neus Barrantes-Vidal, Sergi Ballespí

**Affiliations:** 1 Department of Psychobiology and Methodology of Health Sciences, Universitat Autònoma de Barcelona, 08193 Barcelona, Catalonia, Spain; jaume.vives@uab.cat; 2 Sport Research Institute UAB, Universitat Autònoma de Barcelona, 08193 Barcelona, Catalonia, Spain; 3 Department of Clinical and Health Psychology, Universitat Autònoma de Barcelona, 08193 Barcelona, Catalonia, Spain; cmcmorales.uab@gmail.com (C.M.); Neus.Barrantes@uab.cat (N.B.-V.); 4 Department of Mental Health, Fundació Sanitària Sant Pere Claver, 08004 Barcelona, Catalonia, Spain; 5 Centre for Biomedical Research Network on Mental Health (CIBERSAM), Instituto de Salud Carlos III, 28029 Madrid, Spain

**Keywords:** mentalization, metacognition, impairment, emotional distress, daily life events

## Abstract

The main aim of this paper is to analyze to what extent insight (i.e., mentalization referring to one’s own mental state) moderates recovering from daily life events. A total of 110 participants (84.5% women; mean age: M = 21.5; SD = 3.2) filled in the Trait Meta-Mood Scale (TMMS-24) and the Eysenck Personality Questionnaire (EPQ-R), and were interviewed about impairment derived from daily life events (everyday life stresses) during the past year. Multivariate regression models were adjusted for neuroticism, sex, and socioeconomic status to analyze whether different degrees of insight moderated the relationship between the intensity and the duration of emotional distress. Results showed that the global measure of insight did not moderate recovering from daily-life distress. Regarding the subdimensions, attention to emotional reactions was related to an increased duration of distress. Results showed that, against our hypothesis, deeper comprehension of emotional reactions, operationalized here as “true insight”, was not associated to faster recovery. Limitations and recommendations for further studies are discussed considering these results.

## 1. Introduction

Mentalization (MZ) is the capacity to be aware of the intentional mental states (i.e., intentions, feelings, desires, thoughts) that underpin human behavior [1]. This capacity involves understanding both one’s own mental states as well as those of others, and their connections with behavior [2]. MZ is the umbrella term adopted to systematize the study of a higher-order cognition (HOC) [3], approached from several perspectives during the twentieth century. Thus, terms like social intelligence, social cognition, theory of mind, mind-blindness, alexithymia, inter-personal intelligence, intra-personal intelligence, emotional intelligence, or insight all refer to the capacity of our brain to be aware of our and others’ mental states. The mentalization paradigm systematizes the field from a multidimensional and neuroscientifically-based umbrella approach that encompasses related concepts and reduces term dispersion [1,2]. From this perspective, mentalizing activity can be cognitive or affective, explicit or implicit, based on external or internal cues, and refers to one’s own or to others’ mental states.

### 1.1. Mentalization Referred to One’s Own Mental States and Mental Health

This study is focused on the dimension of mentalizing referring to one’s own mental state. Recent developments suggest that the capacity to be aware of one’s own mental state (1) is impaired in presence of psychopathology, regardless the type of disorder of psychopathology [4,5,6,7,8,9,10,11,12,13,14,15]; (2) is usually preserved in healthy populations, so it probably is an important factor to preserve mental health; (3) has always been an important function to promote in all treatments that work [16], thus suggesting that it is an important dimension to work with in order to recover mental health. Given this, it is assumed that the awareness and comprehension of one’s own mental state plays a role in the metabolism of emotional distress.

### 1.2. Mentalization as a Resilient Capacity to Cope with Daily Life Events

However, there is also the idea that “to see more” (i.e., “to be more aware of” mental states) is not necessarily associated to better mental health [17]. In fact, sometimes a way to cope distress is “not being aware” of disturbing events, because this suppression from consciousness leads to less suffering than being consciously exposed to that content [18,19].

Both the MZ capacities and the individual threshold of distress are associated with individual differences. Referring to this last concept, there are at least two important factors that might be involved in the amount of daily-life emotional disturbance: daily hassles and emotional sensitivity.

Daily hassles or everyday life stresses, as opposed to chronic stressors (e.g., low socioeconomic status, chronic illness, constant work demands) and major traumatic events (e.g., child or adult abuse), are minor disruptions of daily life that challenge psychological well-being, but which are usually associated to relatively rapid recovery. Examples of these “quotidian” stressors or daily life events are low-severity illness, academic evaluations, or current family conflicts that, in spite of not being as challenging as a chronic or a severe illness, prolonged economic problems, or the loss of loved ones, clearly have an impact in daily functioning and emotional well-being [20].

Accordingly, the greater the number of daily hassles or daily life events, and the higher the emotional reactivity (i.e., neuroticism), the higher the daily-life emotional distress. This is a well-established point both in clinical [21] and non-clinical populations [22].

Nevertheless, the question involved in the current study is this: once distress is present in daily life functioning, to what extent does MZ play a role in recovery? That is, we wonder if MZ capacities (and more specifically, insight or MZ referring to one’s own mental state) confer resiliency. Since resiliency means the capacity to “adapt successfully to adversity, involving successful recovery from adverse life events and sustainability in relation to life changes” [23], mentalization can be considered a resilient capacity if it fosters recovery.

### 1.3. The Levels of Emotional Awareness in the Metabolism of Emotional Distress

The aforementioned leads to hypothesis 1, i.e., that MZ referring to one’s own mental state (also referred to as “insight”, “emotional awareness”, “or emotional metacognition”) moderates the relationship between the intensity and the duration of daily-life emotional distress.

Considering that the emotional impact of a stressor or a daily hassle is different for different people, according to individual differences (the importance of or the sensitivity to that specific hassle in each individual, the emotional sensitivity or reactivity of one to daily life events, the emotional situation that one has in that moment of life), and also considering that this “internal” consequence of the hassle (i.e., the emotional consequence that the hassle produces in a person) can be quantified through two indicators of the “amount of pain” (the intensity and the duration of the emotional distress produced [24]), it is to be expected that the higher the intensity of the emotional distress, the longer the duration will be, because the time required for recovering is proportional to the magnitude of damage to recover from; moreover, according to our hypothesis, this relationship is moderated by the capacity to “metabolize” this distress. Therefore, the higher the capacity to mentalize and digest distress, the shorter will be the duration expected for a given magnitude of distress, because MZ catalyzes recovering.

However, considering the debate about the goodness of insight versus the opposite mechanism (i.e., repression, distraction, “out of sight, out of mind” mentality), we aim to go further and to analyze whether different levels of emotional awareness may be differently associated with recovering. Metacognition (also called “intrapersonal intelligence” [25]) is a whole process conceptualized as a sequence of mental functions that starts from attention to emotions and runs to emotional regulation [26]. Emotional awareness, then, is not a simple state, but a process based on progressive achievements [27]. Salovey and colleagues [28] structure the ability of emotional metacognition in three dimensions: attention, comprehension and emotional regulation. Considering that, the true effective action to digest distress (named here “true insight”) is very probably not placed in the first step of that mentalizing process, because simply paying attention to emotions seems to be different from truly comprehending them.

### 1.4. The Different Role of “Simple Attention” versus Comprehension of One’s Own Emotional Reactions

In fact, in light of clinical (e.g., most anxiety disorders, depression) and non-clinical phenomena suggesting that paying excessive attention to one’s own mental state (i.e., emotions, body reactions, distorted thoughts) increases emotional arousal [29] and blocks adaptive behavior [30], our second hypothesis predicts that while a “second degree” of emotional awareness (i.e., comprehension, clarity, emotional recognition,) constitute “true insight”, and therefore might foster recovering as it helps to digest emotional distress (that is, it will attenuate the relationship between the intensity and duration of distress) [12], simple attention to emotional distress (the first step of the MZ process to insight) might be related to increased emotional suffering and slower or incomplete recovering.

In other words, hypothesis 2 argues that while (a) attention to mental state will amplify the positive association between intensity and duration of distress, (b) the comprehension of our own mental state (i.e., a second degree of awareness subsequent to simple attention) will lessen the positive association between the intensity and duration of distress.

This combined hypothesis allows us to unify the idea that insight is indeed resiliency—when we are able to achieve “true insight”, or comprehend beyond simple attention—while simple attention to negative emotion, without comprehension, can be “invasive” and counter-productive, as it just increases emotional arousal.

To delve deeper into this combined concept, we finally aimed to run one more analysis, in order to check whether different combinations of those two steps of meta-mood cognition (first, attention, and then comprehension) are involved in the metabolism of distress. To do that, we converted the dimensional measurements of attention and comprehension processes into binary variables that classify participants into high-capacity (75th percentile scores or higher) or average (scores below the 75th percentile). This allowed us to form four groups of participants according to their high/low insight abilities or mentalization profile.

The group with high attention and high comprehension was composed of those participants with scores above the 75th percentile in both MZ dimensions. This group was referred to as “True Insight” and corresponds to the healthiest MZ option; it was expected to play more resilient-like role in regard to distress. The group with neither high attention nor comprehension was named “Mind-Blinded”, which, considering the aforementioned effect of excessive attention in the absence of comprehension, was not expected to represent the most disadvantaged situation in a context of distress. An MZ profile based on high attention and not-high comprehension is was to lead people to become overwhelmed by increasing emotional arousal without the possibility of digesting it. This group was labelled “Invaded”. Finally, there was a group with non-high attention but high comprehension that showed a kind of automatic MZ capacity (high comprehension without high attention), and its actions would probably be closer to that of the True Insight group than those of the other groups, because both include the important ingredient: comprehension.

Focusing on these groups, hypothesis 3 predicted that the True Insight group would be more associated with a reduced time of distress compared to the Mind-Blinded group, and even more clearly compared to the Invaded group.

## 2. Methods

### 2.1. Participants

The initial sample consisted of 120 participants (83.3% women; mean age = 21.5, SD = 3.2), who were enrolled in the second phase of the Mentalizing Project. The Mentalizing Project aims at analyzing the association between MZ and mental health, and involves the voluntary participation of undergraduates from the Autonomous University of Barcelona. All participants signed an informed consent.

The psychopathological conditions could affect MZ capacities, and for that reason, 10 participants were excluded from the present study. The final sample was composed of 110 participants (84.5% women; mean age = 21.5, SD = 3.3). Of these, 9.1% came from families at a high socioeconomic level, 23.6% from families at a medium–high level, 20% from families at a medium level, 28.2% from families at a medium–low level, and finally, 19.1% from families at a low socioeconomic level, according to the Hollingshead index [31].

### 2.2. Materials

#### 2.2.1. Interview

Based on the most used interviews to assess major traumatic events [32], a simple ad hoc interview was designed to specifically identify how many “minor” stressful events the individual had experienced in the last year from the date of the interview, and how much emotional distress they created, operationalizing this “amount of emotional suffering” as trauma interviews do, according to American Psychiatric Association Criteria [24]—that is, based on intensity of distress (i.e., quantity or degree of negative emotion) as well as the duration of the emotional reaction.

This interview was comprised of a first part for screening psychopathology, based on conventional, semi-structured clinical interviews, as well as a second part to assess daily life events. In order to anchor the retrospective assessment from the present to the past, participants were first asked about their current level of well-being or happiness in the last week from the date of the interview, as well as their usual mean level of happiness, both using Likert scales from 1 (minimum happiness) to 10 that participants directly rated. Using the mean level of emotional well-being as a reference, they were then interviewed about those situations or events from the last year that impacted in some manner in their usual level of emotional well-being, even minor situations (i.e., a low mark in an exam, a cold).

For each episode, participants were asked about how they felt (i.e., sad, angry, nervous, frustrated), in order to register the kind of descriptors they used to refer emotional suffering. Then they were asked to rate the intensity of the emotion (“How sad did you feel in that moment from 1, minimum sadness, to 10, maximum?”) using a Likert of 9 points (1–10), and the duration of the emotional distress in weeks. Note that participants were interviewed on the base of the slumps of their usual well-being (i.e., “Tell me when you felt worse than usual, emotionally speaking; when do you remember being less happy than you usually are?”) to then ask about the reason (the external event) and the amount of emotional suffering (intensity and duration of the emotional reaction). Participants who indicated some type of severe mental disorder, such as eating disorders, autolytic aggressions, or generalized anxiety disorder (*n* = 10), were excluded.

#### 2.2.2. TMMS-24

The Trait Meta-Mood Scale is a measure of perceived emotional intelligence in terms of individuals’ beliefs about their own emotional intelligence. It consists of three subscales (attention, comprehension, and regulation) with eight positively worded items each. Items concerning the “attention” domain evaluate the degree to which individuals notice and think about their feelings; items in the “comprehension” subscale refer to the ability to understand and recognize one’s mood; items concerning the “regulation” domain evaluate the degree to which individuals moderate and adjust their moods and emotions [28]. Each item is rated on a five-point Likert scale (e.g., 1: Do not agree at all; 3: Agree pretty much; 5: Agree completely). The internal consistency of TMMS-24 attention and comprehension subscales was measured in the current sample with Cronbach’s alpha, obtaining, respectively, coefficients of 0.91 and 0.90.

#### 2.2.3. EPQ-R Version

The Revised Eysenck Personality Questionnaire (Spanish version) evaluates the three basic dimensions of personality: extraversion (scale E), emotivity (scale of neuroticism, or N), and hardness (scale of psychoticism, or P). It also has a fourth scale: compliance (L scale). It is a paper and pencil test of individual and collective application from the age of 16. The EPQ-R has 83 items in the version adapted by the Spanish population. Cronbach’s alpha of the E, N, P, and L scales in the current sample ranged from 0.71 to 0.86.

### 2.3. Procedure

The present study is part of the Mentalizing Project. Data were gathered from a self-selected sample of participants who agreed to participate in the study. Participants were first asked to respond to the TMMS-24 [33], EPQ-R [34], and Hollingshead index [31] over the internet. Participants were invited to the lab where, after signing the informed consent, they were interviewed to evaluate the intensity and duration of distress over the last 12 months, as well as perform a screening of their psychopathology.

### 2.4. Statistical Analysis

The sample size was calculated using G*Power 3.1.9 (Heinrich-Heine-Universität Düsseldorf, Germany)) [35]. For a small to medium size effect (*f*^2^ = 0.1, α = 0.05, power (1-β) = 0.8), with two explanatory variables and two control variables, the sample needed was 100.

Linear regressions were conducted using IBM SPSS Statistics v25.0 package (Armonk, NY, USA). To study Hypotheses 2a and 2b, attention and comprehension factors were entered, and each two-way interaction was tested first. Following a backwards model selection strategy, we first removed from the model the non-significant interaction parameters.

All models tested met the assumptions of independent errors, homoscedasticity, and absence of multicollinearity. As the assumption of the normality of residuals was barely met, *p*-values and confidence intervals are based on a bootstrap of 1000 samples with a bias-corrected accelerated (BCa) method. Results are presented as linear regression coefficients (*b*), reporting bootstrap with 95% confidence intervals (95% CI) and *p*-values (*p*). All regression models were tested controlling for sex, socioeconomic status (SES), and neuroticism (EPQ-R). SES did not enter the final model, due to an irrelevant effect on regression coefficients. All regression coefficients were adjusted for sex and neuroticism (EPQ-R).

## 3. Results

According to our first hypothesis, MZ referring to our own mental states (called “insight”) moderates the relationship between the intensity and the duration of daily-life emotional distress. Given this, a multiple linear regression was performed, with global intensity of distress (GID) as the exposure variable, global duration of distress measured in weeks (GDD) as the response variable, and the global measure of insight as a moderator. Results show that a global measure of insight does not moderate the duration of distress (*b* = 0.008; 95% CI = −0.022–0.36; *p* = 0.51). In fact, even in a model of main effects (i.e., assuming no moderation effect), insight is not significantly related to the duration of distress.

In contrast to Hypothesis 2a, the results reveal that comprehension of mental states does not moderate the relation between GID and GDD (*b* = 0.18; 95% CI = −0.99–1.04; *p* = 0.7).

**Figure 1:**
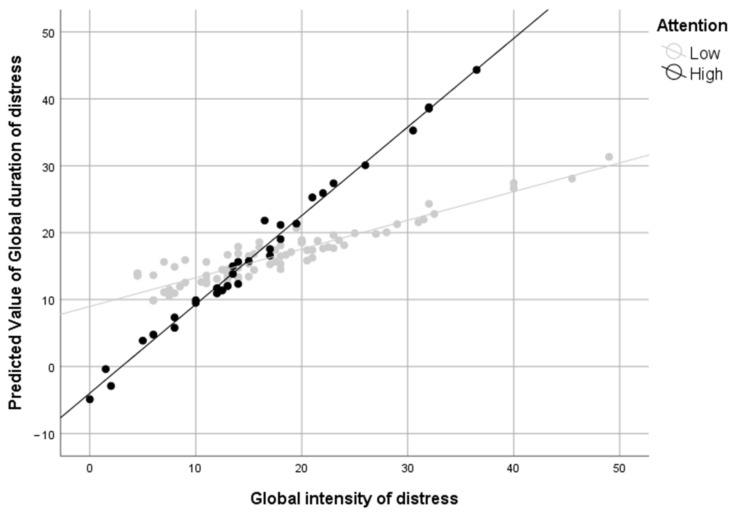
Predicted values of global duration (weeks) of distress from global intensity of distress and attention, controlling for sex and neuroticism.

However, our results do support Hypothesis 2b, as there is a statistically significant moderation of attention and GID. The effect size (see Table 1) of this moderation indicates that the relationship between GID and GDD varies depending on attention, so that for every unit of increase in intensity of distress, the duration of distress increases between 0.1 and 1.6 (95% CI) units more when there is high attention than when there is not. This moderation effect is depicted in Figure 1.

**Figure 2:**
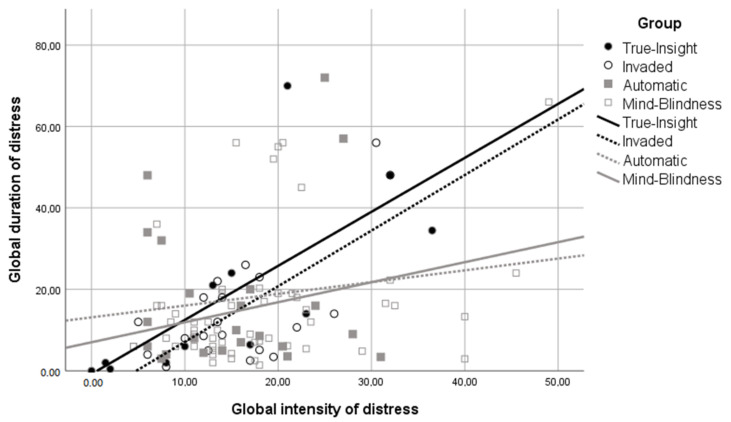
Grouped scatterplot of global duration (weeks) of distress and global intensity of distress.

Finally, when different combinations of high/low dimensions of insight are tested, and in contrast to Hypothesis 3, no significant differences were obtained between the four groups. Figure 2 (which does not include adjustment for the control variables) suggests that it is attention and not comprehension, independently of the value of the other capacity, which might play a moderating role on distress duration. This figure shows that the lines including high attention (black lines with/without dashes; pertaining to the True Insight and Invaded groups), independent of the level of comprehension, have a steeper slope than lines representing groups with non-high attention (grey lines with/without dashes, pertaining to the Invaded and Automatic groups), independent of high or low comprehension. This suggests, once more, that attention to emotional reactions is associated with an expected increase in the duration of distress.

## 4. Discussion

The global aim of the present study was to analyze to what extent insight, a higher-order cognition that implies being aware of one’s own mental state [3], fosters recovering from daily hassles. To our knowledge, this is the first study to analyze the moderator role of mentalizing in the recovery from daily hassles in a sample from the general population.

Our findings reveal that insight, as a global measure based on the Trait Meta-Mood Scale, is not significantly associated the duration of daily-life distress, nor does it moderate the relationship between the intensity of daily-life distress and its duration. According to this result, insight does not provide the “emotional digestive action” attributed to this mental capacity, and suggests that if emotional awareness does not foster recovery, then the recommendations from popular culture (“out of sight, out of mind”) and some cognitive behavioral therapy techniques [36] are probably more useful.

However, this first analysis included a global measure of meta-cognition. Therefore, it was still possible that a separate analysis of the subprocesses included in the global measure (attention, comprehension, regulation) would shed light on this issue. In this sense, only partial support for the second hypothesis was obtained: although attention moderates the duration of distress in the predicted direction, comprehension, the expected active ingredient [17], does not play the predicted role attributed to the True Insight group, nor does it foster recovery.

The last analysis did not reveal differences regarding the moderation effect of different MZ profiles on distress duration. In fact, no MZ profile showed a significant moderation effect on the duration. It is interesting to observe the results when control variables are excluded, as shown in Figure 2, it suggests that attention, independent of the presence or absence of the next step to “true insight” (comprehension), is associated with an increase in the duration of distress.

Overall, these findings not only do not support the idea of insight as an emotional metabolism factor, but rather they go against this thesis. Three analyses with different approaches led to similar conclusions: attention to emotional reactions is associated with an increased duration of distress, thus making sense of the classic recommendation of “out of sight, out of mind”. The idea that comprehension constitutes “true insight” and that it is the best option to deal with distress was not supported here.

This is surprising, because there are several reasons to derive this hypothesis: (1) insight is generally impaired in the presence of psychopathology, thus suggesting that it can be a cornerstone of mental health [4]; (2) this is why most psychological treatments implicitly or explicitly work on insight to foster recovery [16,17]; (3) acceptance of health conditions or consciousness of mental state and illness are important processes, even in health recovery, and they have a role in important intermediate processes like adherence to treatment, trusting in the therapists and treatment or the rapport [37]; and (4) even in non-clinical population, the awareness of one’s own inner world has been pointed as a resilient process [38]. Therefore, insight is important to recover mental health and probably to maintain it, and people with good insight seem to show better functioning [39]. Thus, we wonder why something largely debated throughout history and quite strongly supported from theoretical, clinical, and even empirical points of view, is not supported here.

The current study has some limitations that may explain some of the results, at least partially. First, although data from daily hassles and emotional distress was gathered through an interview, the measure of insight was self-reported. This is important because a self-report measure of insight can be affected by the lack of insight, and beyond the interview that a clinician can conduct with a well-known patient, or the kind of interview usually used in research to assess mentalization, based on the analyses of the transcription of an extensive attachment interview, which is subsequently scored to assess global (not only self) mentalizing capacity of the participant, there is no workaround this shortcoming. That is to say, there is no standardized procedure, alternative to an extensive interview, to measure self-mentalization or insight in the same way as mentalizing about others’ mental states is measurable, as far as we are concerned.

In fact, as an additional shortcoming, the retrospective estimation of distress could also be influenced by memory biases, defense mechanisms, and MZ variations. This means that people with more defensive MZ profiles perhaps were informed by not having suffered much or having recovered fast, thus minimizing the impact of events and distress. Similarly, maybe people in the Invaded group overinformed the intensity and duration, inflating these measures, since they tend to become overwhelmed by emotion due to an excess of attention without the capacity to digest. Apparently, this might be controlled for by the inclusion of a measure of neuroticism in the model, but non-self-reported measures of insight might be able to refine these results. This means addressing a pending difficult question: how to create a standardized measure of MZ referring to one’s own mental states. Finally, another limitation coming from the sample is that it was self-selected and mainly composed of women. While the analyses in this study are controlled for sex, the characteristics of the sample impose some limitations regarding the external validity of this study.

Further research should also consider other possible approaches. For instance, we here tested how MZ moderates recovering from daily hassles, but do not consider the objective severity of those events. How MZ intervenes once emotional distress is already present was analyzed in the current study, but it is very possible that good MZ also intervenes, moderating the impact of daily life events—that is, influencing (buffering) the intensity with which people experience those events. Furthermore, it might be interesting to analyze the interaction between the two moderator effects, between how mentalizing buffers the stressful effect of the environment and how it fosters recovery.

In this sense, a last reflection should be done. The current approach claims that MZ (specifically, comprehension) fosters recovery because it helps to digest, and according to how we operationalized “better recovery”, a shorter duration was expected, assuming that MZ accelerates digestion, and therefore people spend less time in distress and recover earlier. It is possible that “better” does not mean faster recovery. Maybe the best way to shorten “sensation of distress” is precisely by not paying attention (i.e., repression or distraction: “out of sight, out of mind”). However, this does not necessarily mean a better recovery, only a faster one. Therefore, if true emotional digestion takes time, a different operationalization of “proper emotional metabolism” should be considered in future studies to empirically analyze something widely accepted in the clinical field: the awareness and comprehension of the emotional world is associated with mental health.

## 5. Conclusions

The results of this study do not support that self-mentalizing or insight, that is, attention and comprehension of one’s own emotions, fosters recovering from daily hassles. Against predicted, a global measure of insight was neither associated with a decreased duration of daily-life distress, nor moderated the relationship between the intensity and the duration of daily-life distress. Dissecting insight into two processes that conform it, attention and comprehension of one’s own emotions, provided slightly different results that suggest that it is attention the ingredient that moderates the relation between intensity and duration of distress, and does it in the predicted direction, i.e., the more attention the more effect of intensity of distress in its duration. These results would suggest that avoiding paying excessive attention to distressful daily events is a more effective strategy than spending efforts in trying to process or comprehend them. In any case, future research with bigger representative samples, gathering more objectifiable data based on ecological momentary assessments to avoid recall biases, may shed further light.

## Figures and Tables

**Table 1:** Moderation of attention on the global duration of distress.

	Global Duration of Distress (*n* = 110)	
	*B* (95% CI)	*p*
GID	0.44 (−0.1–0.94)	0.100
Attention	−12.34 (−23.16–−1.81)	0.023
Moderation	0.85 (0.1–1.55)	0.022

GID: global intensity of distress; 95% CI: bias-corrected and accelerated confidence interval; *p*: bootstrap statistical significance. Attention (high vs. low).
